# Childbearing Decision Making: A Qualitative Study of Women Living with HIV/AIDS in Southwest Nigeria

**DOI:** 10.1155/2012/478065

**Published:** 2012-12-20

**Authors:** Y. A. Sofolahan, C. O. Airhihenbuwa

**Affiliations:** Department of Biobehavioral Health, Penn State University, 315 Health and Human Development East, University Park, PA 16802, USA

## Abstract

Using the PEN-3 model, the purpose of this qualitative study was to understand the factors responsible for the childbearing decisions of women living with HIV/AIDS (WLHA) in Lagos, Nigeria. Sixty WLHA who sought care at a teaching hospital in Lagos were recruited to participate in in-depth interviews. The average age of the participants was 30 years, and 48 participants were receiving antiretroviral therapy. Healthcare and spiritual practices, healthcare provider-patient communication about childbearing, and husband/partner support emerged as factors that contribute to the childbearing decisions of WLHA. The findings reveal the importance of discussing sexual reproductive health and childbearing issues with WLHA in the healthcare context prior to pregnancy.

## 1. Introduction

Childbearing (CB) is a source of concern for women living with HIV/AIDS (WLHA), because of the risk of HIV transmission to children and sexual partners [1–4]. WLHA must consider many factors when making childbearing decisions, including support from partners and healthcare providers [1, 3, 5].

As the HIV/AIDS epidemic enters its third decade, the reproductive choices available to WLHA are evolving. The initial recommendations of the CDC in 1985 and the American College of Obstetrics and Gynecology in 1987 discouraged WLHA from getting pregnant [6]. In 1994, the American Society for Reproductive Medicine encouraged physicians to discuss other options such as assisted reproductive technology [6]. Unfortunately, some of the recommended assisted reproductive technologies are not widely accessible to WLHA in resource-constrained settings [4]. However, given that many women believe that a woman's identity is affirmed by her motherhood status [5, 7–9], many WLHA in these settings make plans to have children with partners whose HIV statuses are sometimes unknown. By doing so, WLHA are at an increased risk for infection with other STIs or reinfection with a different strain of HIV by engaging in unprotected sexual practices to become pregnant [1, 4]. 

Women in sub-Saharan Africa between the ages of 15 and 24 years constitute 76% of those at risk for contracting HIV, and the risk of infection for this group is three times that of the general population [10]. Because HIV affects mostly women in their reproductive years, decisions about childbearing among WLHA continue to be a subject of debate in resource-constrained settings. Despite advances in antiretroviral (ARV) therapy and prevention of mother-to-child transmission services, many WLHA in these settings wrestle with the decision to have children [1, 3]. Moreover, since it is perceived that many healthcare workers are unsupportive of WLHA childbearing plans, WLHA often are discouraged from having children [3].

In this paper, we examine the ways in which childbearing decisions of WLHA are influenced, especially by partners, families and healthcare workers [1, 2, 4, 11, 12]. Moreover, the power to make such decisions depends on the information available to these women and how independent or autonomous they are within their families and society at large [7]. Many WLHA in this study population in Nigeria do not have the independence or autonomy to make decisions on childbearing outside their sociocultural norms [7, 13].

Our aim was is examine the childbearing decision making process of WLHA by utilizing the culture-centered PEN-3 model. We assess the values and beliefs that underlie WHLA perceptions; reveal enablers, such as available healthcare support and resources; and identify nurturers, such as the influence of partners involved in their decision making.

## 2. Theoretical Framework

The PEN-3 cultural model is used to examine the role of culture in addressing beliefs and behaviors that contribute to health decisions [7, 14]. The PEN-3 model emphasizes the need to focus on the cultural factors that influence decision making [7]. In other words, the emphasis is not on the individual, but on multiple factors that collectively shape health decisions.

**Figure 1:**
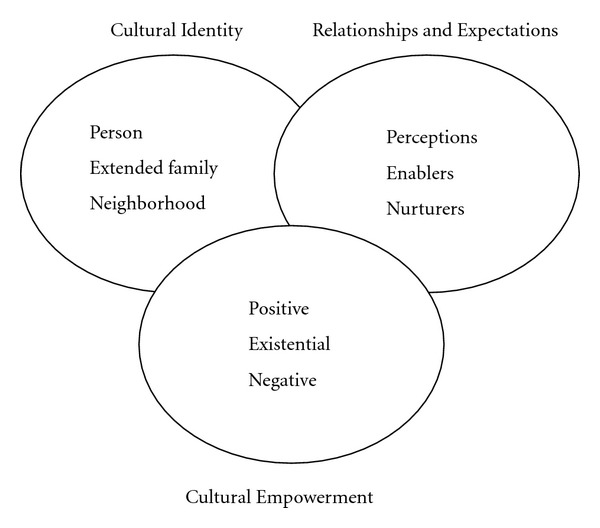
The PEN-3 model.

PEN-3 has three domains, and each domain has three dimensions (see Figure 1). The three interconnected domains are cultural empowerment (CE), relationships and expectations (RE), and cultural identity (CI). CE considers the positive, existential, and negative cultural values that are factored into health behaviors and decisions. RE considers factors such as perceptions, enablers, and nurturers that influence health behaviors and decisions. CI reveals the appropriate level of focus for health interventions—the person, the extended family, or the neighborhood—by addressing how one's identity plays a critical role in influencing health decisions [7, 14].

RE is the domain of interest in this study, which explores the perceptions, enablers, and nurturers that facilitate or hinder childbearing decisions of WLHA. Perceptions include the values and beliefs that may promote or hinder healthy behaviors when factored into childbearing decisions of WLHA. Enablers are the institutional (healthcare) support services that may influence healthy behaviors and practices among WLHA that may affect childbearing decisions. Nurturers are partners and family members who may support or discourage childbearing among WLHA. 

## 3. Methods

### 3.1. Study Site

The study was conducted between July and August 2011 at the hematology clinic of the Lagos State University Teaching Hospital (LASUTH), located in Southwest Nigeria. With a population of about 9 million, a total fertility rate of 5.4%, and a mix of Nigerians from different ethnic groups, Lagos is one of the most populous states in Nigeria [15–17]. The study site was ideal because it provides HIV care and treatment free of charge, which enables WLHA from diverse backgrounds to access care. The clinic also provides free counseling and testing services, as well as HIV support groups.

### 3.2. Study Design

Using a qualitative research design methodology, in-depth interviews were conducted over a 2-month period with 60 WLHA who attended the hematology clinic. A semistructured interview guide adapted from Cooper et al. [1] was used to explore childbearing desires and sexual and reproductive healthcare (SRH) needs, and their influence on the childbearing decisions of WLHA. The first author interviewed participants individually in a private room at the clinic. All interviews were audio recorded and conducted in one of the three main languages spoken in Lagos (English, Pidgin English, or Yoruba). Verbal informed consent was obtained from participants prior to recording. Each interview lasted between 45 and 60 minutes. The participants were given 1000 Naira ($7) as an incentive to cover their transportation costs. Ethical approval was obtained from the Institutional Review Boards of Penn State University and LASUTH.

### 3.3. Recruitment

Purposive sampling was used to recruit WLHA between the ages of 18 and 43 years who were receiving care at the hospital. The first author obtained permission from the department head at the clinic after explaining the purpose of the study and eligibility criteria to the resident physicians. Initially, participants were recruited through referrals from the resident physicians. About a week into the study, however, we realized that physicians often forgot to refer potential participants to the study because the clinic was so busy. In addition, when referrals were made, potential participants were not interested in extending their time spent at the hospital by participating in interviews, as they simply wanted to complete the tasks that brought them to the hospital. So, we devised an alternate approach and recruited potential participants while they were waiting to collect a 3-month supply of ARV drugs at the pharmacy. This approach worked better, because WLHA were more relaxed during the final stage of their visits. Out of the 63 participants recruited, three refused to participate in the study, either because they did not want to be recorded or due to time constraints.

### 3.4. Data Analysis

The first author conducted a preliminary analysis of the transcripts from the first five interviews to determine the aspects of the interview guide that needed to be revised or removed for clarity. All interview transcripts were thoroughly read by the first author to become immersed in the data and then loaded into NVivo 9 to aid in organization and data management. Using constant comparison consistent with Glaser and Strauss' [18] approach to open coding, we generated free nodes. Based on similarities, we then organized these free nodes into related categories or themes guided by the PEN-3 model to generate tree nodes (axial codes). Finally, we organized emerging themes into categories within the relationships and expectations domain of PEN-3. 

## 4. Results

### 4.1. Demographics

Participant demographic information is summarized in Table 1. 

Using the PEN-3 model, the results from our in-depth interviews revealed three themes and two subthemes:

1. the role of faith in perceptions about childbearing decisions;
2. patient-healthcare provider communication as an enabler in child bearing decisions;
3. partner support as a nurturing influence on childbearing decision making, including
  - support informed by knowledge and awareness of HIV,
  - support informed by denial of infected partner's HIV status.

### 4.2. Perceptions in Childbearing Decision Making: Role of Faith

Even though many of the participants held strong spiritual beliefs, almost all of them recognized the importance of utilizing available healthcare services instead of relying solely on spiritual practices such as faith healing. Nevertheless, some of the women felt they had to consider other spiritual alternatives in order to become mothers, since they believed that medical care alone would not result in successful childbearing. While some women believed in a combination of healthcare and spiritual (prayer) practices, others believed in just one or the other. 

One of the most revealing findings that emerged was the perception that HIV is a spiritual problem caused by “evil or wicked forces” that curse a woman, thereby preventing her from becoming a mother. Most of the women who held these beliefs had lost multiple children or had experienced difficulty getting pregnant, even after adhering to the HIV treatment regimens recommended by their physicians. One participant said:

*I believe that somebody that has HIV does not die quickly, that's why I wanted to know why I lose baby after 25 days, because I used my drugs faithfully when I was pregnant and followed what the doctors told me to do. Next time, I will go to the church since this thing may be a spiritual problem and spiritual problems need spiritual solution (28 years old).*

Most women still utilized the services of the clinic during pregnancy, especially for delivery. Many also continued faith healing practices, which they believed would help their children to be born HIV negative. Most acknowledged that faith healing would not cure them of HIV, but they did believe that such practices would cure them of the underlying cause of childlessness, the “evil forces.” 

Some women thought that adhering to ARVs would prevent all medical problems associated with pregnancy. One participant said:

*I was very angry; you know that after all my effort taking the drugs and following all the doctor's advice, I still lost another baby. So, in 2008 when I got pregnant again, I decided to just go to the church for prayers and my ANC (antenatal care). I (went to the hospital) and told the doctor to go through CS (cesarean section), I didn't breastfeed and I was not taking any drugs and my baby is negative. During this pregnancy, I was not going to a hospital. I was just going to the church for prayer because I believe that God will help me break the evil (curse) so I can keep a pregnancy (34 years old).*

When asked if she would do anything different if she were to get pregnant again, she responded:

*If I want to get pregnant, I will be careful. I will follow what they tell me to do here (hospital) and I will also go to church for prayer. Let nurses help me on (what to) do (so) that the baby would not contract HIV again. That is the only thing that I need from them. *

### 4.3. Enabling Factors in Childbearing Decision Making: Patient-Provider Communication

Our interviews revealed that most women wanted healthcare workers to initiate discussions about sexual reproductive health (SRH) and CB. When healthcare workers simply ask if WLHA have any complaints or problems, it does not encourage open discussion about SRH and CB issues. One 27-year-old participant noted, “some people may not have the heart to talk about it… For some people if you don't ask they will not say anything. You ask, “is everything okay?” They say, “okay,” even if it is not.”

Very few WLHA who desire and intend to have children have initiated these conversations with their healthcare providers due to the perceived stigma associated with childbearing among WLHA [19]. WHLA are more likely to initiate these discussions with healthcare workers whom they perceive as supportive of their childbearing goals [1, 20]. WLHA are more likely to open up when healthcare workers ask them specific questions about their childbearing desires and intentions [21]. For many participants, healthcare workers who initiated such discussions enabled them “to open up freely.” One 33-year-old participant remarked, “it is good if they start asking about it (CB) so that many of us can open up and they can advise us.” Another participant added that it is beneficial when healthcare workers initiate these discussions, because

*It will help them (healthcare workers) touch every other part of your life that has to do with this thing (HIV) that most people are shy or don't have the confidence to discuss. If they notice that you are asking them the questions and you are interested… they will open up about their childbearing plans… and use it (the information) to help themselves and things will get better (25-year-old).*

When physicians initiate SRH/CB discussions, WLHA “have the free mind to start telling them about the other (related) things,” which results in better provider-patient dialogue and, potentially, better healthcare experiences.

When healthcare workers did not ask questions related to SRH, some women perceived that such topics were off limits and not to be discussed. One participant noted:

*Well, if perchance during consultation a doctor asks leading questions, then it can prompt you to open up, but where they don't even broach such subjects at all, then there is no way you can open up, because it's like we're here for A and you're talking about B. It's a different thing where a doctor says that even though I know that we're here for A, you can talk about B. Feel free to talk about B, C, and D (34 years old).*

On the other hand, some women saw initiating such conversations as being beyond the scope of healthcare services. Others were unsure of the type of SRH/CB conversations they could have with their healthcare providers or the right time to broach certain topics, particularly given their sensitive and intimate nature. One 25-year-old participant with persistent itching and discharge in her genitals said, “I was thinking in my mind whether I can ask him or show him something like this. Can I tell the doctor something like this?” This sentiment also was expressed by a 28-year-old participant who had recently experienced a miscarriage. When asked if she told her physician about the miscarriage, she explained that she did not, because “if they don't ask you, you will not say.” When healthcare workers do not ask questions related to SRH/CB needs, it is a missed opportunity and a great disservice to WLHA. 

For those WLHA who summon the courage to ask questions about SRH and CB, the advice they usually get from healthcare workers is, “when you are ready, tell us and we will let you know what to do.” In this setting, being ready refers to fulfilling marital and reproductive goals, referred to as* life projects of marriage and reproduction* [8]. For WLHA, being armed with SRH and CB information prior to getting pregnant is essential, since some pregnancies are unplanned. One 27-year-old participant said, “they can also be telling those of us that are not married so that we will know what to do and how to go about it when that time comes.” 

For most participants, the sex of the physician was not a major issue in determining the content of their discussions. However, the physician's approach and interactions with them seemed to matter more in influencing the doctor-patient relationship. A 25-year-old participant said:

*There are some doctors that you meet and the way they welcome you will give you more assurance to open up to him or her. When a person is approaching you like that, you will feel free to open up and your mind will be relaxed. It does not matter to me if it is a male or female doctor.*

In addition, a physician who “shows real interest” and does not see a WLHA “as an object or a figure” will encourage open discussion. Some women noted that supportive and encouraging healthcare workers can make them feel at ease and “alive” when discussing SRH/CB issues.

*Before I open my mouth to tell the doctor that I want to get pregnant, I just read his face. Within that 2 or 3 minutes I read his face to know (that) he is not harsh, and that is what gave me the zeal to ask him questions. When I said I had questions, he said, “oh go on my ears are welcome.” When I now told him, he said, “what are you waiting for (that you have not had another baby)? If na me be your husband, I for dan give you double belle (If I were to be your husband, I would have impregnated you with twins by now).” He was just saying it jokingly and that made me feel comfortable to go ahead and get pregnant. There are some doctors I cannot talk to like that, because of how harsh they are (32 years old).*

### 4.4. Nurturing Influences in Childbearing Decision Making: Role of Partners

Contrary to the negative message in the literature focusing on the nonsupportive role of partners of WLHA, most participants reported that their husbands and partners were supportive. Although the definition and degree of partner support varied, some forms of support were informed by knowledge and awareness of HIV, while others were informed by denial of their partner's HIV status. Support could take on different forms, from the partner “being there” to “encourage,” “advise,” “fight HIV together,” “share each other's burden,” and “console,” to more tangible support, such as going along to the hospital or providing transportation money. 

Most of the women who had disclosed their status to their partners reported that their partners were supportive and saw them as “normal,” and “not as someone who is positive.” This form of support can have potentially negative consequences for their partners. One 28-year-old participant said:

*When I told my husband, he told me to remove my mind from it and I should not think about it (HIV). He is like second god to me. He advises me a lot. Right from the first day, I don't think about it at all and forget there is something like this in me because of his support. I live my life normal, even sleep with my husband normal (unprotected).*

Most women reported being indebted to their partners for the kind of support they received. As such, they were willing to do anything to reward their partners, even engage in unprotected sex. One participant adhered to her medications for this very reason:

*I will allow him to have his fun (sex) with me, and that is why I don't miss my drugs. I know I am not protecting myself only; I am also protecting people around me. If that is what he wants, I will allow him because of the kind of support he has given me (30 years old).*

This sense of indebtedness is driven by the fact that HIV “has broken many homes;” in fact, “there are some women that are having problems at home because of their status.”

Childbearing was central to the support provided by husbands to their wives. Many husbands stated that the reason they stood by their wives was because they wanted to have children. As a way of thanking husbands for their support, WLHA were willing to do whatever it took to have children. One participant expressed fear of losing her marriage, and discussed how she actively showed appreciation to her husband for his support:

*This (children) is what my husband wants and this is what I will give him, because he has been patient and supportive from day one… You don't know their mind at all. All these men can be funny with your status again. And he is negative. Anything can happen to your marriage (32 years old).*

Negative past experiences influenced some women to “secure the relationship” by waiting until they got married and became pregnant before disclosing their status.

*I only told my boyfriend who is now my husband, about my status when I got pregnant, because I had several relationships before him and after I told them, it did not work out. So I had to wait before telling my husband until after I got pregnant (28 years old).*

#### 4.4.1. Nurturing and Support Based on Knowledge and Awareness of HIV

After learning from counselors and support groups at the clinic about HIV and ways to avoid transmission, most women reported that they went back home to educate their partners. After educating their partners, participants often received their full support.

*I told him he doesn't have any problem because I have been using my drugs, and there is a way to have children. They lectured us then I used that lecture to teach him. After that, I brought him to the clinic… for counseling. They talked to him, even the lady counselor was positive, too. She said she got married and had kids after, so he supported me. He did the test and he was negative. Since then my husband supports me fully. We did not tell his family because we don't want family problems (28 years old).*

Another participant explained that after disclosure, her partner expressed his support by wanting to learn more about HIV/AIDS in order to continue with the relationship:

*The only thing he asked me was that, “What do I do? What am I supposed to do as I am the opposite person? Do I run a test? Do I take drugs? Do I do this or that?” I just told him, “be yourself.” He even comes to the hospital with me because he wants to know more about HIV (25 years old). *

Another way in which partners showed their support was by covering for their wives in the presence of his family (her in-laws), specifically about infant feeding practices and mode of delivery. When in-laws started to become suspicious, husbands would step in to dispel any rumors.

*I did not breastfeed my baby at all, and my husband's family had a problem with that. They (in-laws) would call my husband and ask him why I am not breastfeeding. My husband had to lie that because of the CS (cesarean section) I did I can't breastfeed because the child will reject the breast milk, so we have to give her SMA (formula) (28 years old).*

WLHA also reported that they were able to extend the support received from their partners to encourage other WLHA who were in similar situations. A 29-year-old participant described an encounter with a devastated WLHA who had just learned of her HIV-positive status:

*Because of my own experience, I went to her and asked why she was crying. She said she's HIV positive. I said, “is that why you are crying? If you see me on the road, will you know that I am HIV positive?” The woman said, “no. So, you are positive?” I said, “yes.” I told her, “you are not falling sick, you can do things on your own; your health is okay, so why are you crying?” I asked if her husband knew about her status and she said, “yes.” I told her, “if your husband is not giving you problem, and he is negative, then why are you giving yourself problem?”*

A 41-year-old participant also described how she felt when she had just discovered her status and how she is using that experience to help others: “when I discovered, it really weighed me down. I just felt that all was lost. I felt negative about life. But with the help of my husband who supported me, now I can encourage other younger ladies around.”

#### 4.4.2. Nurturing and Support Based on Denial of Infected Partner's HIV Status

Some husbands and partners refused to accept the fact that their partners were infected with HIV. This type of support has potentially negative consequences, since such partners tend to neglect necessary protective measures to prevent disease transmission.

*He is negative and I am positive, but he still doesn't protect himself from me. Any other man that knows his wife is positive and he is negative will use every opportunity to protect himself at all times, but he doesn't do that (25 years old).*

Some women reported that their partners provided support to them, but refused to accept their HIV-positive status, especially those partners who were HIV negative. For example, a 28-year-old participant noted, “when I first knew (of my status), if my husband wants to make love with me I will give him the condom. He will say, ‘No.' He will tell me, ‘you don't have anything like that.'” This form of support could be problematic, because it prevents WLHA from taking necessary precautionary and preventive measures until it is almost too late, as in the case of a 41-year-old participant:

*He was even the one that confused me. He gave me the impression that I didn't have it, because he was negative. He said I should forget about it and rule it out of my mind and that was why I did not start treatment until when I had the crisis. *

## 5. Discussion and Conclusion

### 5.1. HIV Seroconversion in Infants and Pregnant Women

Infants of HIV-positive mothers are at increased risk for HIV infection, and when infants are infected, the disease progresses rapidly [22, 23]. Due to the latency period associated with HIV seroconversion, a child is declared free from pediatric HIV at 1 year of age after repeat testing or 6 weeks after breastfeeding has ended [23]. In addition, maternal seroconversion of HIV status can occur during early pregnancy (<14 weeks), late pregnancy or even postpartum; that is why repeat testing in late pregnancy (32–34 weeks) and postpartum is often recommended for pregnant women [24]. The World Health Organization (WHO) recommends testing of HIV-exposed infants between 4 and 6 weeks of age, and repeat testing at 9 months and 18 months, as well as 6 weeks after cessation of breastfeeding [22, 24].

Our findings expand on previous work highlighting the dynamic and complex nature of childbearing decisions, which are deeply rooted in personal beliefs and support from significant others [3, 4, 25]. Our findings describe the childbearing decision making process for WLHA within a context of competing priorities among the women, their partners, and healthcare workers. A majority of participants desired to have children despite their HIV status. This was due, in part, to *securing the relationship*. Moreover, their partners wanted them to have children as soon as there were physical improvements in their health, whereas healthcare workers recommend waiting for a high CD4 count and a low viral load before commencing childbearing [1, 3, 4].

Participants believed that combining healthcare services with faith healing practices was the best way to achieve favorable childbearing outcomes. Women sought alternate practices when they believed that medicine could not ward off “spiritual forces” or that healthcare practices had failed them. Our results on the role of faith in childbearing complement findings from Adogame [26], although his study did not focus on childbearing, but on how African Pentecostals deal with HIV/AIDS. Our results also confirm previous findings on the role of spirituality in future childbearing [27–29].

Supportive healthcare workers encouraged WLHA to discuss their childbearing plans with them when they were ready to have children [30]. However, not all women in our study discussed their childbearing plans with healthcare workers [1, 4]. Consistent with findings from previous studies, some WLHA resented the information they received from healthcare workers about planning pregnancy and timing unprotected sex [1, 3, 31]. Given that some WLHA viewed pregnancy as “something that just happens,” not discussing SRH/CB issues in the healthcare setting is a cause for concern due to possible implications for access to preventive and treatment services.

Our findings indicate that most partners were supportive of WLHA, and that this support was expressed in many different ways. Partner support encouraged future childbearing and empowered participants to provide emotional support to other WLHA who were discouraged. This finding is contrary to prior findings that WLHA experience negative consequences such as domestic violence, abandonment, and infidelity after disclosing their status to their partners [1, 2]. 

Consistent with other findings, many participants expressed confusion about serodiscordance, leading them to engage in risky sexual behaviors with their partners or fail to access needed treatment [4]. As found bySmith and Mbakwem[32], participants expressed unprotected sex as a marker of partner support and trust. In addition, partners showed their support by becoming “coconspirators” and covering for their wives in the presence of family and friends [32].

This study has some limitations that should be considered. Participants were not randomly selected, and as such, the findings are biased towards WLHA who access healthcare in clinical settings. Therefore, the results should not be generalized, since they are not fully representative of all WLHA. 

Despite these limitations, the results of this study have implications for healthcare providers. Healthcare workers should provide necessary SRH/CB information to all WLHA, whether they are planning to get pregnant or not, so that they can be prepared to make the right decisions. This is important, because not all pregnant WLHA will come to the clinic for antenatal care; some will seek alternative forms of care. If SRH/CB issues are not discussed prior to pregnancy, WLHA may engage in practices that may be harmful to both themselves and their children.

## Figures and Tables

**Table 1:** Characteristics of the study population.

Characteristic	Number
Mean age (range)	30 y (20–43 y)
Interquartile range	6
20–25	8
26–30	23
31–35	20
36–40	6
41–45	3
Education	
None	2
Primary	4
Secondary	26
Higher	28
Employment status	
None	24
Self-employed	17
Employed	15
Volunteer	4
Ethnicity	
Yoruba	22
Ibo	14
Hausa	2
Ishan	6
Delta Ibo	3
Other	13
Relationship status	
Married	30
Widowed	5
Engaged	5
Single	20
Mean no. of years since diagnosis (range)	5 y (1 wk–10 y)
Currently on ARVs	
Yes	48
No	12
Partner status	
Negative	24
Positive	16
Unknown	20
Disclosure to partner	
Yes	38
No	22
Currently living children	
0	26
1	16
2	8
≥3	10
Future childbearing desire	
Yes	48
No	12
